# Platinum(II) *O*,*S* Complexes Inhibit the Aggregation of Amyloid Model Systems

**DOI:** 10.3390/ijms20040829

**Published:** 2019-02-14

**Authors:** Daniele Florio, Anna Maria Malfitano, Sarah Di Somma, Carolin Mügge, Wolfgang Weigand, Giarita Ferraro, Ilaria Iacobucci, Maria Monti, Giancarlo Morelli, Antonello Merlino, Daniela Marasco

**Affiliations:** 1 Department of Pharmacy, University of Naples Federico II, 80134 Napoli, Italy; floriodaniele1@gmail.com (D.F.); giancarlo.morelli@unina.it (G.M.); 2 Department of Translational Medical Science, University of Naples Federico II, 80131 Napoli, Italy; annamaria.malfitano@unina.it (A.M.M.); sarah.disomma@unina.it (S.D.S.); 3 Institute for Inorganic and Analytical Chemistry, University of Jena, 07743 Jena, Germany; carolin.muegge@rub.de (C.M.); wolfgang.weigand@uni-jena.de (W.W.); 4 Department of Biology, Ruhr-University Bochum, 44801 Bochum, Germany; 5 Department of Chemical Sciences, University of Naples Federico II, 80126 Napoli, Italy; giarita.ferraro@gmail.com (G.F.); ilaria.iacobucci@unina.it (I.I.); montimar@unina.it (M.M.); antonello.merlino@unina.it (A.M.); 6 CEINGE Biotecnologie Avanzate s.c.a r.l., University of Naples Federico II, 80145 Napoli, Italy

**Keywords:** amyloid aggregation, platinum complexes, anti-aggregation properties

## Abstract

Platinum(II) complexes with different cinnamic acid derivatives as ligands were investigated for their ability to inhibit the aggregation process of amyloid systems derived from Aβ, Yeast Prion Protein Sup35p and the C-terminal domain of nucleophosmin 1. Thioflavin T binding assays and circular dichroism data indicate that these compounds strongly inhibit the aggregation of investigated peptides exhibiting IC_50_ values in the micromolar range. MS analysis confirms the formation of adducts between peptides and Pt(II) complexes that are also able to reduce amyloid cytotoxicity in human SH-SY5Y neuroblastoma cells. Overall data suggests that bidentate ligands based on β-hydroxy dithiocinnamic esters can be used to develop platinum or platinoid compounds with anti-amyloid aggregation properties.

## 1. Introduction

The mechanism of action (MOA) of Pt(II)-based anticancer agents, among which cisplatin [*cis*-Pt(NH_3_)_2_Cl_2_] is recognized as a progenitor, is well known. It consists of ‘‘DNA platination’’, i.e., the exchange of a chlorido ligand of the first coordination sphere of Pt(II) with a nucleobase of DNA (for example a guanine) [1]. However, these metal compounds can also interact with proteins and peptides. Crystallographic studies have provided detailed information about the structure of the adducts formed in the reaction of proteins with cisplatin [2,3,4,5], *trans*-Pt complexes [6], *cis*-Pt(NH_3_)_2_I_2_ [7], carboplatin [2,3,4,8,9], oxaliplatin [10] and other Pt(II) complexes, like those bearing *O*,*S*-bidentate [11,12] or terpyridine ligands [13]. The ligands coordinated to Pt(II) determine the reactivity of these complexes with proteins and the binding sites. For example, in the interaction with the model protein hen egg white lysozyme (HEWL), cisplatin [3,13,14], carboplatin, *trans*-Pt derivatives [8,13] and *cis*-Pt(NH_3_)_2_I_2_ [8,15] bind His15, oxaliplatin binds Asp119 [16,17], whereas a Pt(II) terpyridine compound binds the side chains of Lys1, Glu7, His15, Arg14 and His15, Lys13, Lys96, Lys97 and Asn93 [13]. These bioinorganic complexes were also investigated in their kinetic and thermodynamic features [14,15,16].

Beyond their use as anticancer drugs, Pt(II) complexes have been studied for a number of activities [17] including antibacterial and antiparasitic purposes. Furthermore, they could act as potential therapeutics for amyloid-neurodegenerative diseases [18,19]. A pioneering study has demonstrated that phenanthroline-based complexes (Pt-phen, phen = 1,10-phenanthroline) coordinate at the His residues of Aβ_1–40_ and act as potent inhibitors of Aβ aggregation and neurotoxicity, restoring the cell viability of primary mouse cortical neurons [20]. Although the interaction between Pt-phen complexes and Aβ is relatively weak, the coordination of these compounds to the peptide significantly alters its aggregation propensity and toxicity profile, as well as its ability to bind Cu(I, II) and Zn(II) [21,22,23].

Several studies have suggested that the potential therapeutic effects of Pt(II) complexes can be tuned by varying the hydrophobicity and charge of the ligands. Indeed, both the inhibition of the fibrillogenesis as well as the arrest of self-recognition during the aggregation process were observed [24].

Platinoid complexes, such as Ru(II), Ir(III) and Rh(III) compounds, are also good inhibitors of amyloid fiber formation [25]. Ruthenium complexes are often alternatives to Pt-based drugs for the treatment of cancer and have been evaluated for their potential applications in neurodegenerative diseases [26,27,28]. Similarly, octahedral Ir(III) and Rh(III) compounds with the same binuclear ligands [25] are able to rescue the toxicity of Aβ_1−42_ in primary cortical neurons [29] and can act as photo-modulators of amyloid-like aggregation [30]. On the other hand, Co(III) Schiff base complexes are able to bind Aβ sequences through His coordination [31] and to promote hydrolysis of peptide sequences [32].

The MOA of both Pt and platinoid complexes is associated with a direct interaction of the metallodrug with the amyloidogenic monomer that determines a rearrangement of its structure to a species less prone to aggregate. In this respect, it is crucial that the metal complexes undergo ligand(s) substitutions with protein residue side chains, such as the imidazole ring of His, which is the main target for platinoid complexes. Thus, the spatial arrangement of labile positions plays an important role. Two labile positions in *cis* conformation on a cyclometalated Pt(II) complex allows targeting Glu and His residues of Aβ [20]; similarly, two labile *cis* coordinated ligands in Pt-phen complexes [33,34] are responsible for the coordination of the side chains of Asp7, His13, and Lys16 for a short Aβ variant encompassing residues 1–16 (Aβ_1–16_) [35]. The presence of aromatic rings in ligands also allows the formation of aromatic interactions (e. g. π-π interactions) with Phe, His and Tyr side chains of Aβ, as demonstrated by the reaction of Pt(phen)Cl_2_ with Aβ_1–16_. Indeed, Pt-phen complexes were found to coordinate the imidazole group of His6 and His14, whereas cisplatin preferentially binds the side chain of Met35 of Aβ [35,36,37].

However, Aβ peptides are not the only targets as amyloid systems. The aggregation of the peptide spanning residues 106–126 of Prion Protein (PrP_106–126_) can be inhibited by its interaction with the Ru(III) complex NAMI-A or by its analogues [38]. Similarly, it has been demonstrated that the amyloid aggregation of human islet amyloid polypeptide (hIAPP) can be inhibited by metal complexes containing homo-dinuclear Ru [39,40] and hetero-multinuclear Pt-Ru systems [41]. In these cases, the binding of metal complexes to hIAPP produces a spontaneous, enthalpy-driven process, due to both hydrophobic interactions and metal coordination.

**Figure 1:**
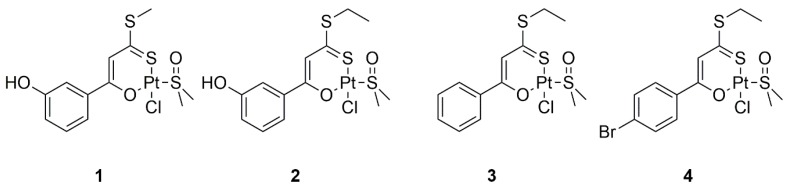
Chemical structures of Pt(II) complexes used in this work.

Here we focus our attention on a series of Pt(II) compounds bearing a conserved *O*,*S* binding moiety, based on β-hydroxy dithiocinnamic esters, as a bidentate ligand (Figure 1). These compounds are appreciably stable in mixed dimethyl sulfoxide−aqueous solvents [11] and cytotoxic for cisplatin resistant cell lines, suggesting a different MOA when compared to cisplatin [42]. To investigate the inhibitory potentials of these molecules on the aggregation of amyloid peptides, we employed three different sequences: (1) the peptide fragment corresponding to the helix H2 (residues 264–277) of C-terminal domain of nucleophosmin 1 (NPM1_264–277_), which shows a remarkable tendency to form amyloid-like assemblies endowed with fibrillar morphology and β-sheet structure toxic to neuroblastoma cells [43,44,45,46,47,48], (2) the heptapetide GNNQQNY, spanning residues 7–13 of the Yeast Prion Protein Sup35p (Sup35p_7–13_), which is involved in the aggregation of Sup35p [49] and (3) the fragment consisting of residues 21–40 of Aβ (Aβ_21–40_) [50]. The three chosen protein fragments are involved in the aggregation mechanism of the related entire proteins.

## 2. Results and Discussion

### 2.1. Pt(II) Complexes with Bidentate Ligands Inhibit the Aggregation of Amyloidogenic Peptides

The ability of the Pt(II) complexes reported in Figure 1 to inhibit the aggregation process of the investigated amyloid peptides was evaluated through fluorescence spectroscopy. Sequences and isoelectric points of the analyzed peptides are reported in Table 1.

Thioflavin T (ThT) binding assay was employed; it is frequently used to analyze the kinetic of the self-recognition process associated with amyloid fibers formation [51].

**Figure 2:**
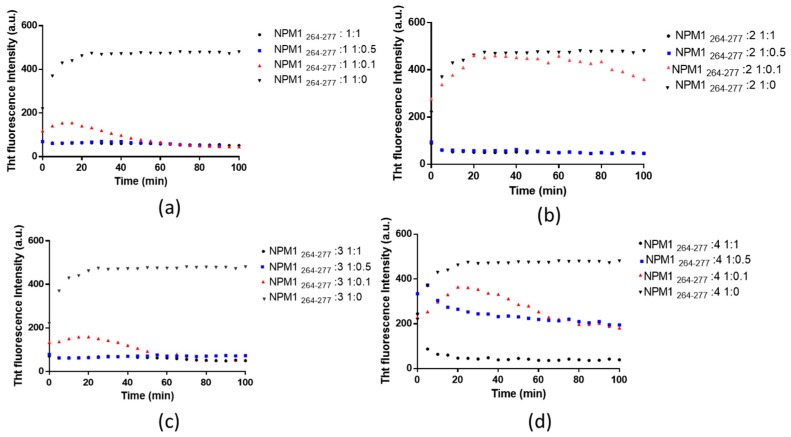
Time course of Thioflavin T (ThT) fluorescence emission intensity of NPM1_264–277_ upon incubation with different concentrations of (**a**) **1**, (**b**) **2**, (**c**) **3**, (**d**) **4**. The peptide alone is reported as a grey triangle. The peptide incubated with the compounds in 1:1, 1:0.5 and 1:0.1 peptide to metal complex molar ratio are reported as a black circle, blue square and red triangle, respectively.

The time course of the ThT fluorescence when NPM1_264–277_ is incubated with the complexes **1**, **2**, **3** and **4** is reported in Figure 2. Fluorescence values were registered for the peptide in the presence of the different concentrations of the Pt compounds, according to the peptide to Pt(II) compound molar ratios indicated in the legends.

NPM1_264–277_ alone, pre-treated with hexafluoroisopropanol (HFIP), exhibits a t_1/2_ value for aggregation of 7 min, as reported in Table 2. When NPM1_264–277_ is treated with the Pt compounds at a molar ratio of 1:1, a clear inhibitor effect is observed. The inhibition of the aggregation is faster for compounds **1** and **3** (Figure 2a,c) than for **2** and **4** (Figure 2b,d). In the case of **1** and **3**, the ThT fluorescence value decreases in less than 5 min, whereas it reduces after 5 min and 20 min in the case of **2** and **4**, respectively. The complete decrease of the fluorescence signal at 481 nm over time of the ThT/NPM1_264–277_ systems in the presence of different Pt complexes suggests that the sample remains in the same monomeric state when treated with Pt compounds at 1:1 equivalents (Table 2).

On the other hand, the anti-aggregation abilities of the investigated compounds are different at lower peptide to Pt complex molar ratios. When NPM1_264–277_ is treated with **1** and **3** at 1:0.1, molar ratio its aggregation is completely inhibited after 50 min. On the contrary, when it is treated with **2** and **4**, the inhibition of the amyloid aggregation is not completed in the investigated time scales. Furthermore, at 1:0.5 peptide to metal complex molar ratio, NPM1_264–277_ provides ThT signals similar to those observed at 1:1 molar ratio in the presence of **1**, **2** and **3**, while it shows a ThT signal that suggests an incomplete inhibition of the aggregation process in the presence of **4**. Potential variations of the fluorescence intensity of ThT caused by Pt(II) complexes are negligible and comparable to the blank signal registered in absence of the complexes.

This analysis indicates that **1** and **3** are the most effective inhibitors for amyloid aggregation of NPM1_264–277_; therefore **1** was chosen for further analyses.

**Figure 3:**
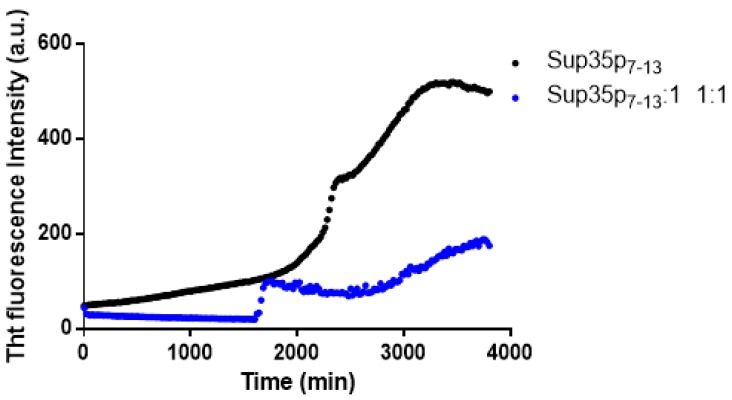
Time course of ThT fluorescence emission intensity of Sup35p_7–13_ (black) upon incubation with **1** at 1:1 peptide to Pt compound ratio (blue).

To verify if **1** could have a similar inhibitory effect on other amyloid systems, the ThT assay was also carried out using Sup35p_7–13_ (Table 1). The time courses of the ThT signals of Sup35p_7–13_, in the presence and in the absence of **1** are reported in Figure 3. The ThT fluorescence intensity of the peptide alone displays two distinct transitions, suggesting a seeding effect of first soluble aggregates to secondly achieve higher levels of oligomerization, as already suggested by other studies [52].

Comparing the ThT signal of the peptide alone with that observed in the presence of **1**, it is clear that the Pt compound affects the aggregation process of Sup35p_7–13_, leading to species with a lower oligomeric state than those found in the case of the peptide alone.

**Figure 4:**
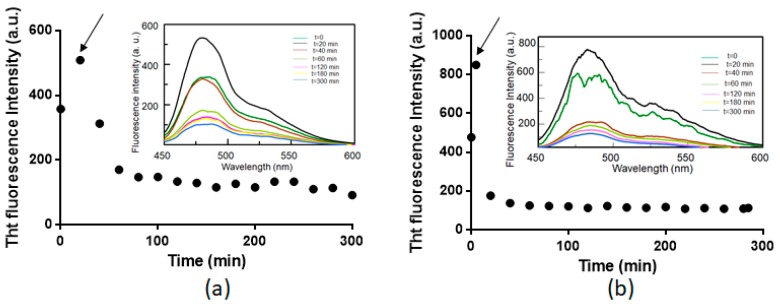
Time course of ThT fluorescence emission intensity of NPM1_264–277_ (**a**) and Sup35p_7–13_ (**b**) upon the addition of **1**. The time of addition of **1** is indicated by an arrow. In the inset, an overlay of the spectra at indicated times are reported.

We have also evaluated the ability of **1** to disaggregate soluble amyloid oligomers, monitoring the ThT signals versus time upon the addition of **1** to NPM1_264–277_ and Sup35p_7–13_ aggregates (Figure 4). The two peptides have different aggregation kinetics, due to their differences in sequence and structure. For this reason, Sup35p_7–13_ was pre-aggregated in the absence of the complexes for one night, whereas NPM1_264–277_ was partially aggregated at *t*_0_ as already reported [43]. Interestingly, we observed a decrease of ThT fluorescence intensity upon the addition of **1** for both peptides. These findings clearly indicate the disaggregating ability of the compound for both amyloid systems.

**Figure 5:**
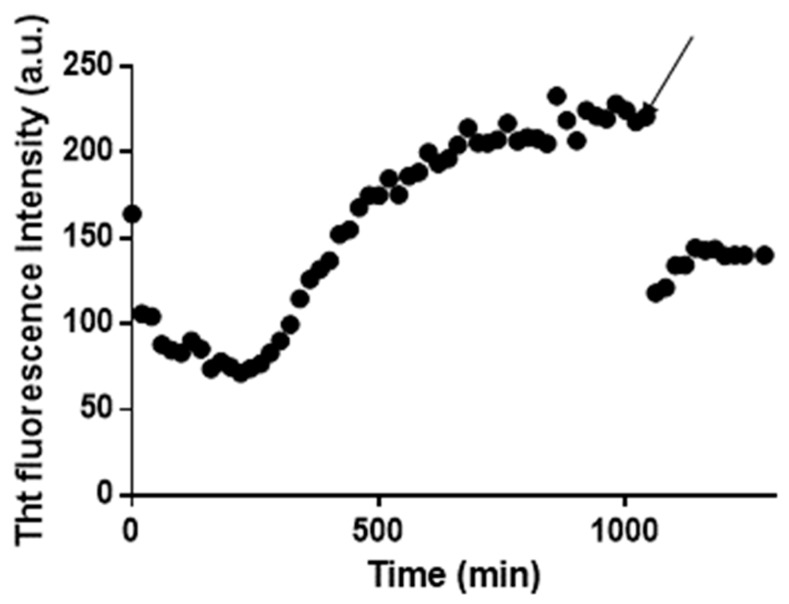
The ThT fluorescence profile of the formation of amyloid aggregates of Aβ_21–40_ and their disaggregation upon the addition of **1** at 1:1 peptide to Pt compound molar ratio. The time of addition of the Pt(II) compound is indicated by an arrow.

A similar experiment was performed using Aβ_21–40_. For this peptide, the ThT fluorescence signal as function of time is reported in Figure 5. The addition of **1** to soluble aggregates of Aβ_21–40_, formed within 1000 min resulted in an instantaneous decrease of the fluorescence intensity, which indicates a disaggregating effect.

**Figure 6:**
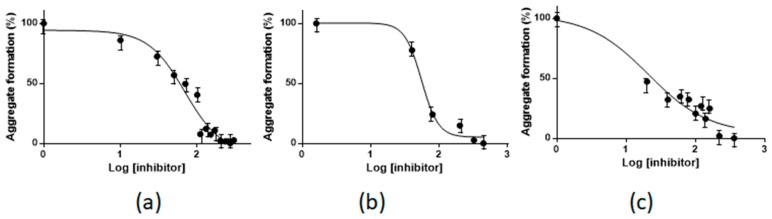
Inhibition of NPM1_264–277_ (**a**), Sup35p_7–13_ (**b**) and Aβ_21–40_ (**c**) aggregation upon the addition of **1**, measured as a percentage variation of ThT fluorescence. IC_50_ values were derived from non-linear regression fit of the data (*r^2^* ≥ 0.98) by fixing the maximum and minimum values at 100 and 0%, respectively, and allowing the Hill slope to vary. Results are representative of two independent experiments.

The ability of **1** to dose-dependently inhibit the aggregation of NPM1_264–277_, Sup35p_7–13_ and Aβ_21–40_ was quantitatively assessed through the comparison of experimental ThT fluorescence values [53], using different metal compound concentrations. The best fittings of experimental data, reported in Figure 6, provide IC_50_ values of 62.3 ± 1.3, 55.03 ± 1.12, 19.9 ± 1.6 µM for NPM1_264–277_, Sup35p_7–13_ and Aβ_21–40_, respectively.

### 2.2. Pt Complexes Inhibit Conformational β-Transition

**Figure 7:**
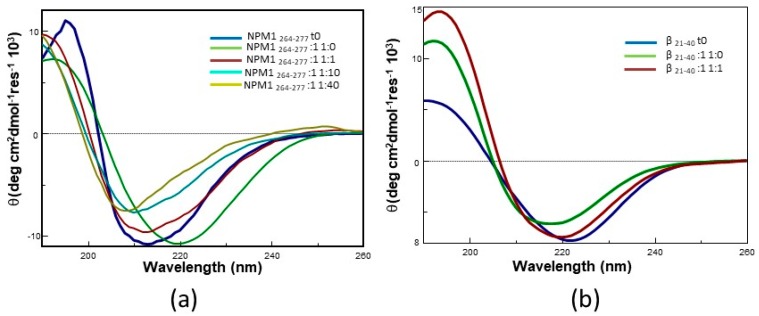
Overlay of CD spectra of (**a**) NPM1_264–277_ and (**b**) Aβ_21–40_ incubated under stirring with **1** at different peptide to Pt compound molar ratio. Incubation time: overnight.

The inhibitory effects of **1** and of the other Pt compounds here investigated could be associated with conformational variations of the analyzed peptides. To study these potential conformational variations, circular dichroism (CD) spectra of NPM1_264–277_ incubated with different equivalents of **1** for one night were compared. CD spectra are superimposed in Figure 7a.

A transition from a mixed α-helix + random coil structure towards a β-sheet structure was previously demonstrated for a variant of NPM1_264–277_ which includes helix 2 and the loop between the 1st and the 2nd helix of the bundle of the C-terminal domain of NPM1 [35].

The spectra of NPM1_264–277_ indicate that, upon overnight incubation, the peptide fibrillates and converts from α-helix to β-sheet (green vs. blue line in Figure 7a). Notably, the presence of the Pt compounds inhibits the α-helix to β-sheet conversion at all the investigated NPM1_264–277_:metal compound molar ratios. Indeed, spectra of NPM1_264–277_ in the presence of the Pt compounds show minima at wavelengths ≤210 nm (Figure 7a), which are diagnostic of the presence of a significant helical content and suggest the formation of ligand-specific secondary structures. A similar behavior has been previously observed when other Pt compounds interacted with Aβ peptides [42].

The same experiment was carried out using Aβ_21–40_. CD spectra of freshly prepared samples of this peptide are already indicative of the presence of a β-structure, as reported in Figure 7b (blue line), thus precluding the possibility to follow the α-helix to β-sheet transition. However, it is interesting to note that the spectrum of the sample corresponding to Aβ_21–40_:**1** in 1:1 molar ratio, after one night of incubation, is more similar to that of the freshly prepared sample of Aβ_21–40_ than to that of Aβ_21–40_ incubated for one night in the absence of **1**.

Attempts to carry out similar experiments using Sup35p_7–13_ failed, since during the aggregation process of this peptide only a significant decrease of the Cotton effect occurred [52] and no substantial differences were observed when the peptide was incubated in presence of **1.**

### 2.3. Mass Spectrometry Analysis

**Figure 8:**
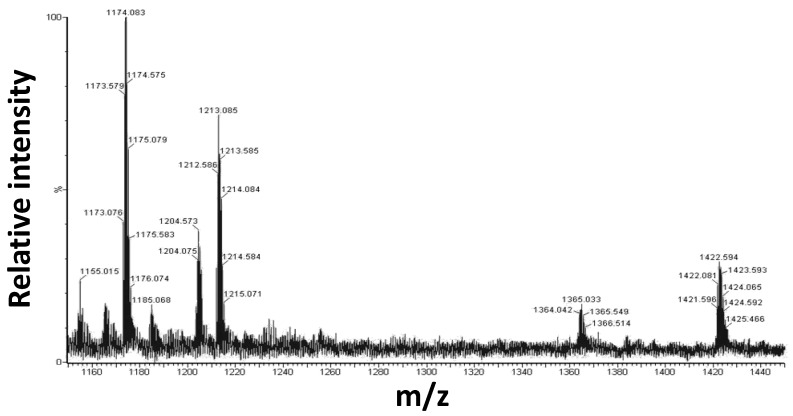
Electrospray ionization mass spectrometry (ESI) spectrum of Aβ_21–40_ with **1**, after 24 h of incubation. All signals are generated from double charged ions.

The peptides and selected Pt compounds (**1** and **3**) at 1:10 ratio, were incubated for 24 h and analyzed by electrospray ionization mass spectrometry (ESI-MS) [54]. As an example, a portion of the spectra obtained for Aβ_21–40_ incubated with **1** is reported in Figure 8.

The presence of double charged signals at 1173.076 and 1212.586 m/z confirms the formation of adducts between Aβ_21–40_ and **1** at 1:1 molar ratio, which are formed upon the release of the chloride and DMSO or solely the chloride, respectively. Furthermore, the signal at 1421.596 m/z corresponds to the double charged peptide ion generated by Aβ_21–40_ bound to 2 Pt complexes, one with the release of the chloride and the other missing a chloride and DMSO. The theoretical and measured molecular masses are reported in Table 3.

Noticeably the sequence NPM1_264–277_ revealed the ability to form adducts with two, three or four Pt derivatives for both compounds **1** and **3.** Results are reported in supplementary Tables S1 and S2.

### 2.4. Inhibition of Cytotoxic Effects of NPM1_264–277_ Peptide in SH-SY5Y Cells

The ability of the Pt(II) complexes to reduce the neurotoxicity of the NPM1_264–277_ peptide was assessed using human SH-SY5Y neuroblastoma cells. Cell survival was evaluated after treating SH-SY5Y cells with the peptide alone or with the mixture of the peptide and Pt(II) complexes.

In comparison with the control sample, the aggregated NPM1_264–277_ peptide showed the highest toxicity at 2 h (cell viability <75%), becoming less effective after 24 h of incubation (cell viability <82%), probably because of the conversion of the early aggregates into larger and less toxic aggregates as already reported for similar amyloids [55] and for NPM1_264–277_ peptide [43].

**Figure 9:**
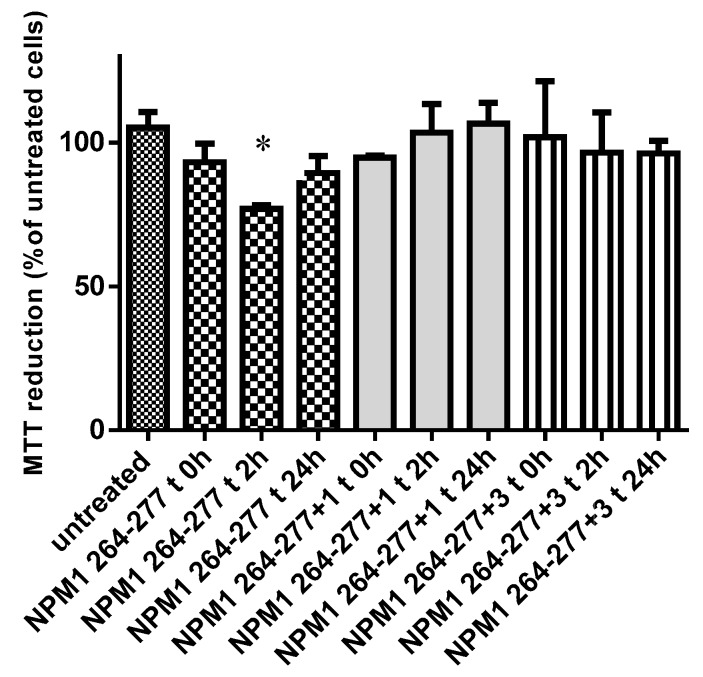
MTT assay in SH-SY5Y cells treated with NPM1_264–277_ and NPM1_264–277_:Pt compounds at 1:10 peptide to metal compounds molar ratio under stirring at three different times, 0, 2 and 24 h, * *p* < 0.05 in statistical analysis.

Compounds **1** and **3** incubated with NPM1_264–277_, at 1:10 molar ratio, seemed to have a protective function against the toxicity induced by the amyloid peptide. Indeed, their presence increased the cell viability values similar to untreated cells (Figure 9), as already reported for similar platinoid compounds [56].

## 3. Materials and Methods

### 3.1. Peptide Synthesis

Amyloid peptides analyzed in this study were synthesized as already reported [35,43]. Their sequences are reported in Table 1. Reagents for peptide synthesis were from Iris Biotech (Marktredwitz, Germany), solvents for peptide synthesis and HPLC analyses were from Romil (Dublin, Ireland); reversed phase columns for peptide analysis and the LC-MS system were from ThermoFisher (Waltham, MA, USA). Peptides’ purity and identity were confirmed by LC-MS. Purified peptides were lyophilized and stored at −20 °C until use. Prior to be analyzed they were all treated for 30 min with HFIP to ensure a monomeric state (at 50% (*v*/*v*) in water), and then the organic solvent was removed by evaporation.

### 3.2. Synthesis of the Complexes

The Pt compounds were synthetized as previously described [11,12]. The stability of the compound under the investigated experimental conditions were tested using UV-Vis absorption spectroscopy, as previously done in ref [11].

### 3.3. Fluorescence Assays

Fluorescence assays were carried out at 25 °C for NPM1_264–277_ and Aβ_21–40_ using the peptide at a concentration of 100 µM and for Sup35p_7–13_ using the peptide at a concentration of 400 µM under the following experimental conditions: 10 mM borate buffer at pH = 9.0, 10% DMSO for NPM1_264–277_ and 10 mM phosphate buffer at pH = 7.4, 0.3% DMSO for Aβ_21–40_ and Sup35p_7–13_. ThT (λ_exc:_ = 440nm, λ_emis_: = 481 nm) fluorescence was measured using a Jasco FP 8300 spectrofluorometer in a 10 mm path-length quartz cuvette, under magnetic stirring. Measurements were collected every 20 min at indicated time intervals. 50 µM ThT was used. Results are representative of two independent experiments.

IC_50_ value was derived from nonlinear regression of the data employing log (inhibitor) vs. response with GraphPad program [57].

### 3.4. Far-UV CD Spectroscopy

CD spectra of NPM1_264–277_ (100 µM, 10 mM borate buffer), Sup35p_7–13_ (400 µM, 10 mM phosphate buffer), Aβ_21–40_ (100 µM, 10 mM phosphate buffer), were recorded on a Jasco J-815 spectropolarimeter (JASCO, Tokyo, Japan), using a 0.1 cm path-length quartz cuvette and different amounts of **1** dissolved in CH_3_CN. The Pt complex was incubated with the peptide for one night; organic solvent was removed through vacuum-evaporation. CD spectra were acquired in the far-UV region and processed as already reported [43].

### 3.5. Cell Culture

Human SH-SY5Y neuroblastoma cells (A.T.C.C., Manassas, VA, USA) were cultured in DMEM, supplemented with 10% FBS, 1.0 mM glutamine and antibiotics. Cell cultures were maintained in a 5.0% CO_2_ humidified atmosphere at 37 °C and grown until they reached 80% confluence for a maximum of 20 passages.

### 3.6. MTT Reduction Assay

NPM1_264–277_ (400µM) peptide was incubated in 50 mM borate buffer under stirring and samples at 1:10 peptide to ligand molar ratio were retrieved at four different times: 0, 2 and 24 h. These were then diluted into cell culture media at a 100 μM, and then added to SH-SY5Y cells seeded in 96-well plates for 24 h at 37 °C. Cell viability was then assessed by the 3-(4,5-dimethylthiazol-2-yl)-2,5-diphenyltetrazolium bromide (MTT) reduction assay as previously described [43].

### 3.7. ESI MS Analysis of the Complexes

Solutions of NPM1_264–277_, Sup35p_7–13_ and Aβ_21–40_ at a concentration of 100 µM in 10 mM borate buffer at pH = 9.0, 10% DMSO for NPM1_264–277_ and 10 mM phosphate buffer at pH = 7.4, 0.3% DMSO for Aβ_21–40_ and Sup35p_7–13_ were diluted 10 times in ammonium acetate 15 mM pH = 7 and analyzed on a Q-ToF Premier (Waters, Milford, MA, USA) by direct injection into the ESI source at a flow of 10 μL/min. The source parameters were set as follows: capillary voltage = 3.6 kV and cone voltage = 42 kV. The acquisition range was set between 600 and 2500 m/z. All data were processed by using MassLynx 4.1 software (Waters, Milford, MA, USA).

## 4. Conclusions

In conclusion, we have studied the capability of Pt(II) complexes bearing *O*,*S* bidentate ligands to inhibit the aggregation process of three different amyloidogenic peptides. The results indicate that the *O*,*S* bidentate ligands of new Pt or Platinoid complexes are promising compounds able to inhibit aggregation of small model amyloid systems. Future studies on full-length proteins should confirm the anti-aggregation properties of these Pt(II) compounds and their potential application as drugs in neurodegenerative diseases.

## Figures and Tables

**Table 1:** Peptide sequences analyzed in this study.

Peptide	Sequence	pI
NPM1_264–277_	VEAKFIN**Y**VKNCFR	9.2
Sup35p_7–13_	GNNQQN**Y**	5.5
Aβ_21–40_	AEDVGSNKGAIIGLMVGGVV	4.5

**Table 2:** Aggregation kinetics (*t*_½_) and maximum fluorescence intensities of NPM1_264–277_ following ThT emission at indicated peptide:Pt compounds molar ratios.

	Peptide to Pt Compound Ratio	*t*_1/2_ (min)	Fluorescence Intensity (Arbitrary Unit)
NPM1_264–277_		5	568
NPM1_264–277_:**1**	1:0.1	6.5	155
NPM1_264–277_:**1**	1:0.5	n.d.	45
NPM1_264–277_:**1**	1:1	n. d.	45
NPM1_264–277_:**2**	1:0.1	7	410
NPM1_264–277_:**2**	1:0.5	n.d.	49
NPM1_264–277_:**2**	1:1	n.d.	48
NPM1_264–277_:**3**	1:0.1	6	155
NPM1_264–277_:**3**	1:0.5	n.d.	70
NPM1_264–277_:**3**	1:1	n.d.	64
NPM1_264–277_:**4**	1:0.1	10	363
NPM1_264–277_:**4**	1:0.5	n.d.	220
NPM1_264–277_:**4**	1:1	n.d.	43

**Table 3:** Results of the ESI-MS analysis of the adducts formed by Aβ_21–40_ and **1**. The experimental m/z values, the ion charge status, the experimental and theoretical monoisotopic mass values and the corresponding ion species are reported.

Experimental m/z, Charge	Experimental Monoisotopic Mass (Da)	Theoretical Monoisotopic Mass (Da)	Pt(II)-Peptide Complexes
1173.056, +2	2344.15	2346.39	Aβ _21–40_ + 1 × (**1**) − 1Cl − 1DMSO
1212.586, +2	2422.16	2424.52	Aβ _21–40_ + 1 × (**1**) − 1Cl
1421.596, +2	2841.16	2844.90	Aβ _21–40_ + 2 × (**1**) − 2Cl − 1DMSO

## Supplementary Materials

Supplementary materials can be found at http://www.mdpi.com/1422-0067/20/4/829/s1.

## Abbreviations

Aβ_1–16_	The peptide encompassing residues 1–16 of the Aβ peptide
Aβ_21–40_	The peptide encompassing residues 21–40 of the Aβ peptide
cisplatin	*cis*-Pt(NH_3_)_2_Cl_2_
CD	circular dichroism
hIAPP	human islet amyloid polypeptide
H2	Helix 2 (residues 264–277) C-terminal domain of nucleophosmin 1
HFIP	hexafluoroisopropanol
MOA	Mechanism of action
NPM1	Nucleophosmin 1
NPM1_264–277_	Residues of helix 2 of Nucleophosmin 1
Pt-phen	Phenanthroline-based Pt compounds
phen	1,10-phenanthroline
PrP	Prion protein
Sup35p	Yeast Prion Protein
Sup35p_7–13_	Residues 7–13 (sequence:GNNQQNY) of Sup35p
ThT	thioflavin T
