# Supplementary material for: Platinum(II) O,S Complexes Inhibit the Aggregation of Amyloid Model Systems
Source: Int J Mol Sci. 2019 Feb 14;20(4):829. doi: 10.3390/ijms20040829 (PMC6413125; doi:10.3390/ijms20040829)
Supplement: Supplementary file 1 [file ijms-20-00829-s001.pdf]

Supplementary to:

Platinum(II) O,S complexes inhibit the aggregation of amyloid model systems

The occurrence of a Cys residue in the NPM1$_{264-277}$ sequence leads to the partial formation of a dimer through a disulphide bridge after 24 h. Also dimeric species gives rise to adducts with Pt(II) complexes.

Table S1. The results of the ESI-MS analysis of NPM1$_{264-277}$ in the presence of **1**. The experimental and theoretical monoisotopic mass values and the corresponding ion species are reported. The species reported without the errors occur as double charged only.

Experimental monoisotopic mass (Da)	Theoretical monoisotopic mass (Da)	Pt(II)-Peptide Complexes
2607.65±0.44	2611.78	NPM1$_{264-277}$+ 2 x (**1**) - 2Cl - 2DMSO
3107.16±1.73	3110.30	NPM1$_{264-277}$+ 3 x (**1**) - 3Cl - 2DMSO
3141.08	3145.75	NPM1$_{264-277}$+ 3 x (**1**) - 2Cl - 2DMSO
3182.26±1.52	3188.43	NPM1$_{264-277}$+ 3 x (**1**) - 3Cl - 1DMSO
3527.22±1.89	3530.69	NPM1$_{264-277}$+ 4 x (**1**) - 4Cl - 3DMSO
3560.44±0.56	3566.14	NPM1$_{264-277}$+ 4 x (**1**) - 3Cl - 3DMSO
3600.87±0.99	3608.82	NPM1$_{264-277}$+ 4 x (**1**) - 4Cl - 2DMSO
3639.7±1.74	3644.27	NPM1$_{264-277}$+ 4 x (**1**) - 3Cl - 2DMSO
3679.33±0.88	3679.72	NPM1$_{264-277}$+ 4 x (**1**) - 2Cl - 2DMSO
5029.65±1.80	5028.33	NPM1$_{264-277}$ dimer + 3 x (**1**) - 1Cl - 1DMSO
5450.99±2.22	5448.72	NPM1$_{264-277}$ dimer + 4 x (**1**) - 2Cl - 2DMSO
5529.12±1.89	5526.85	NPM1$_{264-277}$ dimer + 4 x (**1**) - 2Cl - 1DMSO

Table S2. The results of the ESI-MS analysis of NPM1$_{264-277}$ in the presence of **3**. The experimental and theoretical monoisotopic mass values and the corresponding ion species are reported. The species reported without the errors occur as double charged only.

Experimental monoisotopic mass (Da)	Theoretical monoisotopic mass (Da)	Pt(II)-Peptide Complexes
2604.11	2607.84	NPM1$_{264-277}$+2 x(**3**) -2 Cl - 2 DMSO
3020.11±0.01	3026.26	NPM1$_{264-277}$+3 x (**3**) - 3 Cl - 3 DMSO
3056.10	3061.71	NPM1$_{264-277}$+ 3 x (**3**) -2 Cl - 3 DMSO
3098.63±0.55	3104.35	NPM1$_{264-277}$+ 3 x (**3**) - 3 Cl - 3DMSO
3134.09	3139.84	NPM1$_{264-277}$+ 3 x (**3**) - 2 Cl - 2 DMSO
3438.05±0.05	3444.68	NPM1$_{264-277}$+ 4 x (**3**) - 4 Cl - 4 DMSO
3474.05±0.04	3480.07	NPM1$_{264-277}$+ 4 x (**3**) - 3 Cl - 4 DMSO
3515.08±0.07	3522.81	NPM1$_{264-277}$+ 4 x (**3**) - 4 Cl - 3 DMSO
3551.57±0.41	3558.26	NPM1$_{264-277}$+ 4 x (**3**) - 3 Cl - 3 DMSO
